# Supplementary figures and images for: The lectin-specific activity of Toxoplasma gondii microneme proteins 1 and 4 binds Toll-like receptor 2 and 4 N-glycans to regulate innate immune priming
Source: PLoS Pathog. 2019 Jun 21;15(6):e1007871. doi: 10.1371/journal.ppat.1007871 (PMC6608980; doi:10.1371/journal.ppat.1007871)

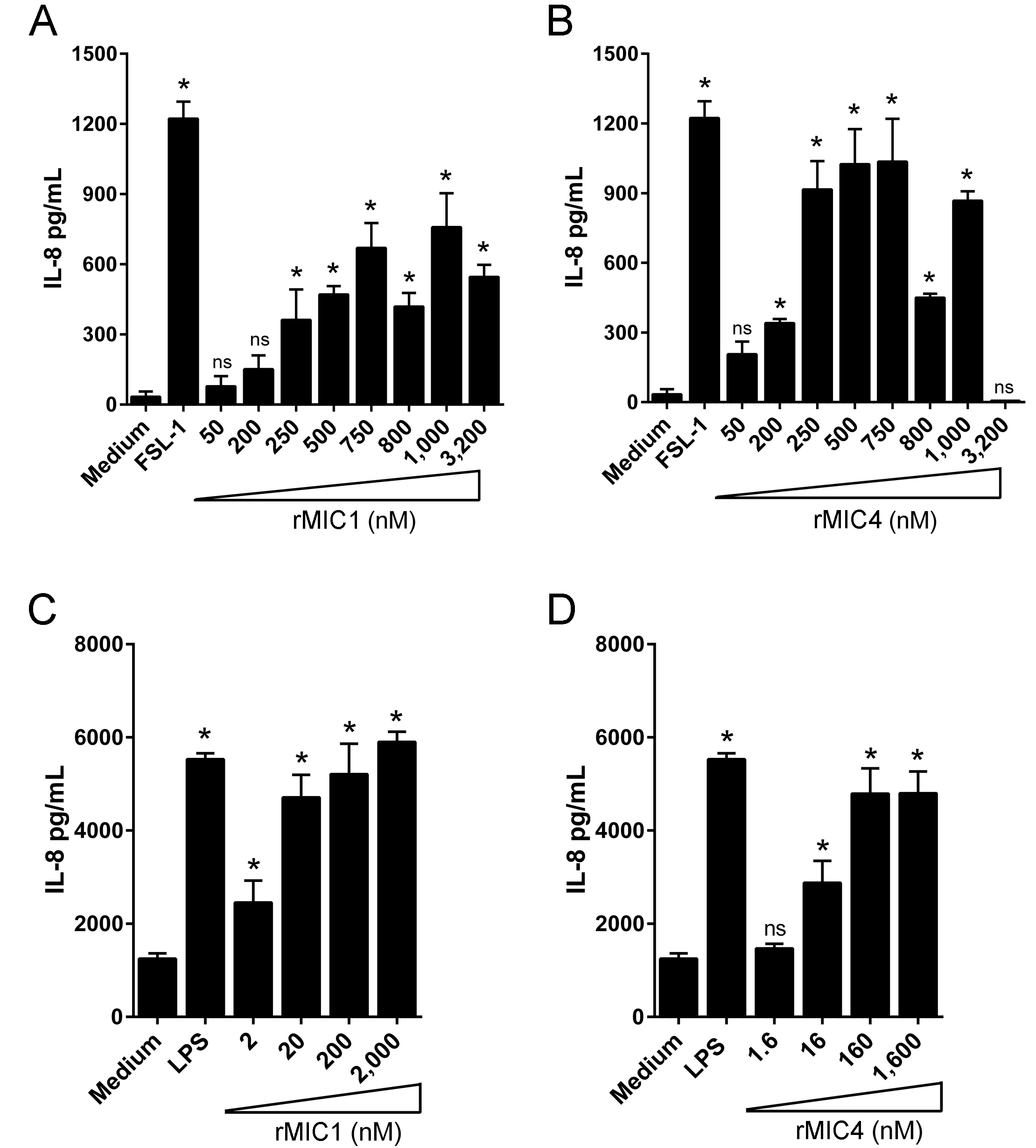

Supplement: S1 Fig — HEK293T cells expressing (A and B) TLR2 or (C and D) TLR4 were stimulated with increasing concentrations of (A and C) rMIC1 and (B and D) rMIC4 for 24 h. FSL-1 (100 ng/mL) LPS (100 ng/mL) were used as positive controls. IL-8 levels were measured by ELISA. Data are expressed as mean ±S.D. of triplicate wells and significance was calculated with ANOVA. *p<0.05. Data are representative of two independent experiments. (TIFF) [file ppat.1007871.s001.tiff]
